# Testing for goodness rather than lack of fit of continuous probability distributions

**DOI:** 10.1371/journal.pone.0256499

**Published:** 2021-09-10

**Authors:** Stefan Wellek

**Affiliations:** 1 Department of Biostatistics, CIMH Mannheim, Mannheim Medical School of the University of Heidelberg, Mannheim, Germany; 2 Department of Medical Biostatistics, Epidemiology & Informatics, University Medical Center of the Johannes Gutenberg University Mainz, Mainz, Germany; Politecnico di Torino, ITALY

## Abstract

The vast majority of testing procedures presented in the literature as goodness-of-fit tests fail to accomplish what the term is promising. Actually, a significant result of such a test indicates that the true distribution underlying the data differs substantially from the assumed model, whereas the true objective is usually to establish that the model fits the data sufficiently well. Meeting that objective requires to carry out a testing procedure for a problem in which the statement that the deviations between model and true distribution are small, plays the role of the alternative hypothesis. Testing procedures of this kind, for which the term tests for equivalence has been coined in statistical usage, are available for establishing goodness-of-fit of discrete distributions. We show how this methodology can be extended to settings where interest is in establishing goodness-of-fit of distributions of the continuous type.

## 1 Introduction

Goodness-of-fit tests belong to the oldest and most frequently used methods of statistical inference. A chapter devoted to them can be found in almost every textbook for statisticians working in whatever area of application. Likewise, there are numerous authoritative expositions of the mathematical theory of these methods, beginning with Cramér’s classical text [1], through all three editions of Lehmann’s “Testing Statistical Hypotheses” [2–4], to Volume 2A of “Kendall’s Advanced Theory of Statistics” [5], to mention just a few highly influential references of this category. Virtually all inferential procedures presented in the existing literature as tests of goodness-of-fit, share one crucial feature: The statement that the model to be fitted coincides with the true distribution from which the data are taken, plays the role of the null hypothesis, implying that a significant result actually indicates lack rather than goodness of fit of the model. This is clearly at variance with the fact that in the vast majority of applications, interest will be in proving rather than falsifying the model so that such a test typically fails to serve the purpose of its user. For instance, many of the most widely used methods of statistical analysis rely on the assumption that the data follow a specific distributional law (namely the Gaussian), and it is widespread practice to make sure of the adequacy of this assumption in a preliminary test. In the latter, the hypothesis one aims to establish states that the corresponding model holds at least approximately true. In the existing literature, the term lack-of-fit test occurs quite infrequently. The main exception is research on methods to be used for detecting misspecifications of linear or generalized linear regression models (see. e.g., [6–9]).

Not surprisingly, the discrepancy between a test of goodness-of-fit and a procedure enabling one to establish the respective model, is addressed at least implicitly in some of the classical expositions of the topic (see, e.g., [5], §§25.6–7). The usual recommendation for finding a way around that difficulty is to take steps for increasing the power of the test, preferably, since increasing the order of magnitude of the sample size will rarely be feasible, by checking the *p*-value against an increased threshold, e.g. 10 instead of 5 percent and deciding in favor of goodness-of-fit if the test does not reject the null hypothesis even at that increased level of significance. However, it is a basic fact that “inverting” a test of a given null hypothesis *H*_0_ against some alternative *H*_1_ by declaring *H*_0_ to be statistically proven if it cannot be rejected, fails to produce a test controlling the risk of taking an erroneous decision in favor of *H*_0_. A procedure which is tailored for serving the latter purpose (“proof of the null hypothesis”), is what has become quite popular in biostatistics since the last few decades under the name equivalence test. Construction of a test of that kind requires to enlarge *H*_0_, before defining it as the new alternative hypothesis, through introducing some indifference zone around the respective point in the parameter space consisting of models deviating from the model of interest by an amount considered still acceptable. The basic requisites for reformulating the testing problem in that way are a suitable measure of distance between true and hypothesized model (often called a metric in a mathematically not fully precise terminology) and a numerical specification of the maximum tolerable value of that distance (called equivalence margin in biostatistical contexts).

Up to now, equivalence tests for goodness-of-fit problems have been made available for problems of establishing models for discrete distributions (see [10], Ch. 9). In Section 2, we develop a framework for equivalence testing in settings where the objective is to establish goodness-of-fit of some hypothesized continuous distribution (like the standard normal law) to the true distribution underlying a dataset under analysis. In the proposed hypotheses formulation, the indifference zone around the model to be established, consists of all Lehmann alternatives [11] to the corresponding cumulative distribution function (cdf) for which the ratio *θ*, say, between the value of the true and the hypothetical cdf at any point in the sample space, falls in a sufficiently narrow interval around unity. Except for considering cdf’s rather than survivor functions which give the probabilities in the right-hand tail of a distribution, the Lehmann parameter *θ* coincides with what plays, under the term ‘hazard ratio’, a prominent role in statistical survival analysis. In Section 3, a uniformly (in *θ*) most powerful, exact test for a hypothesis of this form is derived and shown that its power does only depend on the Lehmann parameter *θ*, not on the cdf one aims to fit to the data. In Section 4. results of studying the UMP test by means of exact numerical methods are presented, focusing on comparisons with tests being available for grouped data taken from the distribution to be fitted. The question of how to extend the approach to settings where the model to be established involves unknown parameters (like location and scale), will be addressed in Section 5. An illustrating example is presented in Section 6.

## 2 Assumptions and hypotheses formulation

Throughout we assume that the assessment of goodness-of-fit of the distribution of interest can be based on a random sample *X*_1_, …, *X*_*n*_ of size n∈N of mutually independent observations. The common distribution of these observations is assumed to be of the continuous type, with *F* as the cdf. The cdf of the distribution to be fitted will be denoted by *F*_0_ and assumed to have support on some (maybe unbounded) interval on the real line. A reasonable basis for constructing a region in the space of all continuous cdf’s on the real line which can be considered equivalent (i.e., sufficiently similar) to the cdf *F*_0_ specified under the model of interest, consists of including all Lehmann alternatives *F*(⋅) = [*F*_0_(⋅)]^*θ*^ for which the maximum vertical distance between *F* and *F*_0_ does not exceed some suitably chosen margin *δ* > 0. Making this idea precise, we start with defining equivalence of the hypothesized to the true distribution by the condition

$$\begin{array}{c} \|F-F_{0}\|\;\equiv \;\underset{-\infty <x<\infty }{\text{sup}}\left|F\left(x\right)-F_{0}\left(x\right)\right|<\delta \;\;\text{for}\;\text{some}\;(\text{fixed})\;\delta >0\;. \end{array}$$ (1)

Modifying the proof given in [12] for survival rather than distribution functions in the straightforward way, it can be shown that for Lehmann alternatives, the condition (1) is equivalent to

$$\begin{array}{c} |\theta^{1/(1-\theta )}-\theta^{\theta /(1-\theta )}|<\delta \;. \end{array}$$ (2)

From basic properties of the expression on the left-hand side of (2) as a function of *θ*, we can eventually conclude that the goodness-of-fit criterion (1) is satisfied if and only if there holds

$$\begin{array}{c} {\left(1+\varepsilon \right)}^{-1}<\theta <1+\varepsilon \;\text{for}\;\text{some}\;\text{suitable}\;\varepsilon >0. \end{array}$$ (3)

Given the equivalence margin *δ* to ||*F* − *F*_0_||, the corresponding value of *ε* is obtained by solving the equation:

$$\begin{array}{c} {\left(1+\varepsilon \right)}^{-1/\varepsilon }-{\left(1+\varepsilon \right)}^{-(1+\varepsilon )/\varepsilon }=\delta \;. \end{array}$$ (4)

Table 1 shows the values of *ε* determined from Eq (4) for a selection of customary specifications of *δ*, together with the right-hand limit of the equivalence range for log *θ* which one is used to consider as the parameter of interest in the proportional hazards model for survival distributions [13].

**Table 1:** Numerical correspondence between equivalence margins referring to ||*F* − *F*_0_|| and the Lehmann parameter *θ*.

*δ*	0.05	0.075	0.10	0.15	0.20	0.25
*ε*	0.1457	0.2266	0.3135	0.5077	0.7341	1.0000
log(1 + *ε*)	0.1360	0.2042	0.2727	0.4106	0.5505	0.6931

**Fig 1:**
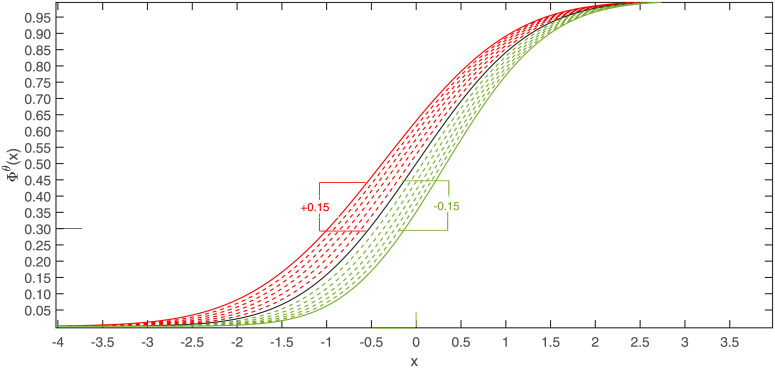
Region of univariate cdf’s being equivalent to Φ (solid line) in the sense of not exceeding a maximal vertical distance of *δ* = 0.15 from the model to be established.

Fig 1 visualizes the equivalence region of the proposed form for the standard normal cdf Φ in the space $F=\{F\;|\;F:R\to \left[0,1\right],F=\Phi^{\theta },\theta \in \left(0,\infty \right)\}$ for *δ* = 0.15.

## 3 A uniformly most powerful test for establishing goodness-of-fit in the continuous case

Adopting the conceptualization of the notion of goodness-of-fit of a specified distribution with cdf *F*_0_ to a sample from an unknown continuous univariate distribution proposed in the Introduction and letting the indifference zone around the model consist of Lehmann alternatives to *F*_0_, we need a test for the problem

$$\begin{array}{c} H:\theta \leq 1/(1+\varepsilon )<\;\text{or}\;\;\theta \geq 1+\varepsilon \;\text{vs.}\;K:1/(1+\varepsilon )<\theta <1+\varepsilon \;. \end{array}$$ (5)

In deriving an optimum solution to this problem, we will make use of the following

**Lemma 1**. Let the *X*_*i*_, *i* = 1, …, *n* be i.i.d. with continuous cdf [*F*_0_(⋅)]^*θ*^ and let

$$\begin{array}{c} {\hat{\theta }}_{n}\left(F_{0}\right)=-n/\sum_{i=1}^{n}\text{log}\left[F_{0}\left(X_{i}\right)\right]\;. \end{array}$$ (6)

Then, the distribution of ${\hat{\theta }}_{n}\left(F_{0}\right)$ does not depend on *F*_0_, and its cdf is, for any *θ*, exactly given by $t\to 1-F_{\chi_{2n}^{2}}(2\theta n/t)$, where $F_{\chi_{2n}^{2}}\left(\;\cdot \;\right)$ denotes the cdf of a central chi-square distribution with *ν* = 2*n* degrees of freedom.

*Proof*. Due to the well-known basic property of the probability-integral transform, we can write for any *i* ∈ {1, …, *n*}: $-\text{log}\;F_{0}\left(X_{i}\right)\;\overset{d}{=}-\text{log}\;U_{i}^{1/\theta }=\theta^{-1}\text{log}\;\left(1/U_{i}\right)$, with $U_{i}∼U\left(0,1\right)$ and $\overset{d}{=}$ denoting equality in distribution. The distribution of log(1/*U*_*i*_) has cdf

$$\begin{array}{c} P\left[\text{log}\left(1/U_{i}\right)\leq \tilde{u}\right]=P\left[U_{i}\geq e^{-\tilde{u}}\right]=1-e^{-\tilde{u}}=G_{1,1}\left(\tilde{u}\right)\;\forall \;\tilde{u}\geq 0\;, \end{array}$$ (7)

with *G*_1,1_(⋅) denoting the cdf of a gamma distribution with parameters (1, 1).

$$\begin{array}{cc} \Rightarrow \;P_{\theta }^{X}\left[{\hat{\theta }}_{n}\leq t\right] & =P_{\theta }^{X}[\sum_{i=1}^{n}-\text{log}F_{0}\left(X_{i}\right)\geq n/t]\\ & =P_{\left(0,1\right)}^{U}[\theta^{-1}\sum_{i=1}^{n}\text{log}\left(1/U_{i}\right)\geq n/t]\\ & =P_{\left(0,1\right)}^{U}[\sum_{i=1}^{n}\text{log}\left(1/U_{i}\right)\geq \theta n/t]\\ & \;\overset{\left(7\right)}{=}1-G_{n,1}\left(\theta n/t\right)=:1-F_{\chi_{2n}^{2}}\left(2\theta n/t\right)\;,\;t>0\;. \end{array}$$

The following proposition states that there is an optimum test for (5) and describes the computational steps to be taken in order to carry out that procedure.

**Proposition 1**. Let the common distribution of the *X*_*i*_ under *θ* = 1 be absolutely continuous with density *f*_0_(⋅). Then, there exists a uniformly most powerful test for (5) which rejects the null hypothesis *H* of relevant deviations of the true from the hpothesized distribution if and only if it turns out that

$$\begin{array}{c} C_{n}^{1}\left(\alpha ,\varepsilon \right)<-n/\sum_{i=1}^{n}\text{log}\left[F_{0}\left(X_{i}\right)\right]<C_{n}^{2}\left(\alpha ,\varepsilon \right)\;. \end{array}$$ (8)

The critical constants $C_{n}^{1}\left(\alpha ,\varepsilon \right)$, $C_{n}^{2}\left(\alpha ,\varepsilon \right)$ have to be determined through solving the equations

$$\begin{array}{c} \;F_{\chi_{2n}^{2}}(2{\left(1+\varepsilon \right)}^{-1}n/c_{1})-F_{\chi_{2n}^{2}}(2{\left(1+\varepsilon \right)}^{-1}n/c_{2})=\alpha \\ \;=F_{\chi_{2n}^{2}}(2\left(1+\varepsilon \right)n/c_{1})-F_{\chi_{2n}^{2}}(2\left(1+\varepsilon \right)n/c_{2})\;,\;0<c_{1}<c_{2}<\infty \;. \end{array}$$ (9)

*Proof*. For absolutely continuous *F*_0_, $X_{i}∼F_{0}^{\theta }$ ∀*i* implies that the joined density $g_{\theta }^{\left(n\right)}$, say, of the sample (*X*_1_, …, *X*_*n*_) is given by

$$\begin{array}{c} g_{\theta }^{\left(n\right)}\left(x_{1},\ldots ,x_{n}\right)=\theta^{n}\prod_{i=1}^{n}{\left(F_{0}\left(x_{i}\right)\right)}^{\theta -1}f_{0}\left(x_{i}\right)=\theta^{n}\cdot \text{exp}\{\theta \sum_{i=1}^{n}\text{log}F_{0}\left(x_{i}\right)\}\cdot \prod_{i=1}^{n}\frac{f_{0}\left(x_{i}\right)}{F_{0}\left(x_{i}\right)}\;. \end{array}$$

Thus, $g_{\theta }^{\left(n\right)}$ is an element of a 1-parameter exponential family, with $\sum_{i=1}^{n}$ log *F*_0_(*X*_*i*_) as a sufficient statistic for *θ*. Hence, a well-known theorem on the existence of UMP tests for equivalence hypotheses about parameters of families of that structure (cf. [10], Appendix A.1) applies, according to which a UMP level-*α* test for (*H*, *K*) has rejection region

$$\begin{array}{c} C=\{\left(x_{1},\ldots ,x_{n}\right)\in R^{n}|(\sum_{i=1}^{n}\text{log}\;F_{0}\left(x_{i}\right))\in \left({\tilde{c}}_{1},{\tilde{c}}_{2}\right)\}\;, \end{array}$$

where $\left({\tilde{c}}_{1},{\tilde{c}}_{2}\right)$ solves the equations

$$\begin{array}{c} P_{\theta_{1}}[(\sum_{i=1}^{n}\text{log}\;F_{0}\left(X_{i}\right))\in \left({\tilde{c}}_{1},{\tilde{c}}_{2}\right)]=\alpha =P_{\theta_{2}}[(\sum_{i=1}^{n}\text{log}\;F_{0}\left(X_{i}\right))\in \left({\tilde{c}}_{1},{\tilde{c}}_{2}\right)]\;, \end{array}$$ (10)

with *θ*_1_ = 1/(1 + *ε*), *θ*_2_ = 1 + *ε*. In view of ${\hat{\theta }}_{n}\equiv -n/\sum_{i=1}^{n}\text{log}\;F_{0}\left(X_{i}\right)$, Lemma 1 implies that a unique solution of (10) is obtained by setting ${\tilde{c}}_{\nu }=-n/C_{n}^{3-\nu }\left(\alpha ,\varepsilon \right)$, *ν* = 1, 2, with $C_{n}^{k}\left(\alpha ,\varepsilon \right)$, *k* = 1, 2, being defined as in (8), (9).

**Remark 1**. The optimal critical constants $C_{n}^{1}\left(\alpha ,\varepsilon \right)$, $C_{n}^{2}\left(\alpha ,\varepsilon \right)$ are not given explicitly but have to be calculated from (9) by means of a suitable numerical algorithm. All results to be presented in the subsequent sections were obtained by means of the program provided as Supplementary Material under the name UMPTestforGoF both as a SAS/IML- and R-script.

**Remark 2**. In terms of both the algorithm for determining the critical constants and its power, the UMP test given by (8) is completely distribution-free. The cdf *F*_0_ defining the model whose goodness-of-fit one wants to establish, is only used for computing the test statistic.

**Remark 3**. Transforming the sufficient statistic $\sum_{i=1}^{n}$ log *F*_0_(*X*_*i*_) to the equivalent statistic ${\hat{\theta }}_{n}\left(F_{0}\right)=-n/\sum_{i=1}^{n}\text{log}\;F_{0}\left(X_{i}\right)$ in writing down the decision rule of the UMP test, is simply a matter of conceptual convenience: ${\hat{\theta }}_{n}\left(F_{0}\right)$ is easily shown to be the ordinary ML estimator of the Lehmann parameter *θ* and thus a quantity straightforward to interpret.

## 4 Numerical results on the UMP test for goodness-of-fit

Table 2 gives a tabulation of the critical values and the power against the null alternative *θ* = 1 of the exact UMP test derived in Section 3 for three different choices of the equivalence margin *ε* and sample sizes *n* ranging from 10 to 200. As usual, the significance level *α* is chosen to be 0.05 throughout. Comparing the entries in different lines of the same block of the table reveals the effect of increasing the sample size on basic characteristics of the test: The left- and right-hand limit of the critical interval which has to be checked for inclusion of the observed value of the ML estimator ${\hat{\theta }}_{n}\left(F_{0}\right)$, is monotonically de- and increasing, respectively, in *n*, in a way making the corresponding intervals a nested sequence of sets. Furthermore, as is mandatory for any reasonable test for the problem put forward in (5), the power increases likewise monotonically in *n* to unity. The effect of increasing the equivalence margin *ε* becomes obvious from comparisons between homologous entries in the different blocks of the table. As has clearly to be expected, the critical interval is shrinking in length as *ε* decreases, and the power attainable with a given sample size declines fairly rapidly as the margin is tightened.

**Table 2:** Critical constants and power of the UMP test at level *α* = 0.05 to detect perfect fit of the model [↔ *θ* = 1] for various sample sizes and choices of the equivalence margin.

*ε*	*n*	$C_{n}^{1}\left(\alpha ,\varepsilon \right)$	$C_{n}^{2}\left(\alpha ,\varepsilon \right)$	*POW*(1)
0.3135	10	0.9836	1.0423	0.0723
"	20	0.9830	1.0427	0.1043
"	50	0.9593	1.0671	0.2919
"	100	0.9047	1.1227	0.7183
"	200	0.8587	1.1737	0.9727
0.5077	10	0.9821	1.0767	0.1142
"	20	0.9560	1.1039	0.2496
"	50	0.8510	1.2125	0.7869
"	100	0.7883	1.2887	0.9857
"	200	0.7481	1.3473	1.0000
0.7341	10	0.9629	1.1450	0.2118
"	20	0.8670	1.2444	0.5738
"	50	0.7400	1.3946	0.9743
"	100	0.6854	1.4822	0.9998
"	200	0.6504	1.5496	1.0000

Recalling the standard approaches to testing for lack-of-fit of a fully specified distributiion to that underlying a given dataset, it seems natural to compare the new test with a goodness-of-fit testing procedure which uses grouped data. In the context of lack-of-fit testing, it is often recommended (see, e.g., [5], §25.22) to choose for grouping classes of equal probability in terms of the distribution *F*_0_ under assessment. Focusing on the best known and perhaps most interesting special case that the distribution one aims to fit is the standard normal N(0,1) so that there holds *F*_0_ = Φ, and that the number *k*, say, of classes to be formed equals 5, a partition of the range of *X* which is in line with that recommendation, is given by the intervals (−∞, −0.8416], (−0.8416, −0.2533], (−0.2533, 0.2533], (0.2533, 0.84162], (0.84162, ∞). If the equivalence margin for the Lehmann parameter is chosen to be *ε* = 0.5077 (corresponding to a maximal allowable vertical distance of *δ* = 0.15 between Φ^*θ*^ and Φ), the probability masses, say $\pi_{j}^{\left(1+\varepsilon \right)}$, of these intervals under *θ* = 1 + *ε* are computed to be

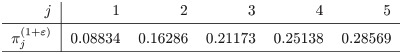


The goodness-of-fit test for multinomial distributions established in Ch. 9.1 of [10] defines equivalence between multinomial distributions of common dimension *k* in terms of the Euclidean distance between the corresponding parameter vectors ***π*** and ***π***°, where ***π*** refers to the unknown distribution underlying the data and ***π***° to the model to be fitted. With the grouped-data probem under consideration, we have $\pi_{j}^{{}^{\circ}}=1/5$ ∀ *j* = 1, …, 5, and the Euclidean distance of the vector ***π***^(1+ *ε*)^ with the above listed components from ***π***° is computed to be *d*(***π***, ***π***°) = ε_*_ = 0.1548. On the other hand, ***π***^(1+*ε*)^ is the parameter vector of the multinomial distribution into which the uniform distribution on {1, …, 5} generated from N(0,1) by means of the chosen partition, is mapped through moving *θ* to the (right-hand) boundary of its equivalence range. Thus, it seems reasonable to consider the problem

$$\begin{array}{c} H_{*}:d\left(\pi ,\pi^{{}^{\circ}}\right)\geq \varepsilon_{*}\equiv 0.1548\;\text{versus}\;K_{*}:d\left(\pi ,\pi^{{}^{\circ}}\right)<0.1548 \end{array}$$ (11)

concerning the parameter ***π*** of a multinomial distribution over {1, …, 5}, as the grouped-data analogue of the testing problem to which the results shown in the middle block of Table 2 relate. As shown in Wellek, loc. cit., an asymptotically valid test of (11) is given by the following decision rule:

$$\begin{array}{c} \text{Reject}\;H_{*}\Leftrightarrow \;d^{2}\left(\hat{\pi }\text{,}\;\pi^{{}^{\circ}}\right)<\varepsilon_{*}^{2}-u_{1-\alpha }\;v_{n}\left(\hat{\pi }\text{,}\;\pi^{{}^{\circ}}\right)/\sqrt{n}\;, \end{array}$$ (12)

where

$$\begin{array}{c} v_{n}^{2}\left(\hat{\pi }\text{,}\;\pi^{{}^{\circ}}\right)=4[\sum_{j=1}^{k}{\left({\hat{\pi }}_{j}-\pi_{j}^{{}^{\circ}}\right)}^{2}\;{\hat{\pi }}_{j}-\sum_{j_{1}=1}^{k}\;\sum_{j_{2}=1}^{k}\left({\hat{\pi }}_{j_{1}}-\pi_{j_{1}}^{{}^{\circ}}\right)\left({\hat{\pi }}_{j_{2}}-\pi_{j_{2}}^{{}^{\circ}}\right){\hat{\pi }}_{j_{1}}{\hat{\pi }}_{j_{2}}]\;, \end{array}$$ (13)

and $\hat{\pi }$ denotes the vector of relative frequencies of observations falling in the different classes. For small to moderate sample sizes, the power of the grouped-data goodness-of-fit test given by (12) and (13) against the null alternative ***π*** = ***π***° can be computed even exactly. With *k* = 5, ***π***° = (1/5, …, 1/5), ε_*_ = 0.1548, one finds that the power values computed for the exact UMP test with the non-grouped continuous data reduce to

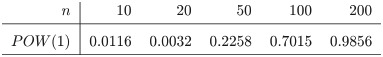


Thus, the fact being well known (see. e.g., [14], Ch.27) for the lack-of-fit case that grouping entails substantial losses in efficacy, has also to be stated for problems of testing for goodness-of-fit.

## 5 A glance at options for testing for goodness-of-fit of distributions involving nuisance parameters

Upon noticing the results presented in Sections 3 and 4, it seems natural to ask the question whether the approach admits generalization to settings where one aims to fit a distribution involving unknown nuisance parameters rather than being fully specified. The best known special case of such a problem is testing for normality, which is to say that the hypothesis of interest states that, except for differences one is willing to accept, the distribution underlying the data has cdf $F=F_{0}(\frac{\cdot \;\;-\mu }{\sigma })$, with *F*_0_ = Φ and arbitrary (*μ*, *σ*) ∈ $R\times R_{+}$. Adapting the hypothesis formulation proposed in the case of a fully specified distributional model to this setting is straightforward, leading to consider the testing problem

$$\begin{array}{c} H:\left(\theta ,\mu ,\sigma \right)\in ((-\infty ,1/(1+\varepsilon )]\cup [1+\varepsilon ,\infty ))\times R\times R_{+}\\ \;\text{versus}\;K:\left(\theta ,\mu ,\sigma \right)\in (\left(1/\left(1+\varepsilon \right),1+\varepsilon \right))\times R\times R_{+}\;. \end{array}$$ (14)

As before, *θ* denotes the Lehmann parameter inducing potential deviations of the true distribution from the distribution of the form assumed under the model to be fitted.

A promising and often successful approach to the construction of equivalence tests in multi-parameter families of distributions uses the maximum likelihood estimator of the parameter of interest as pivotal quantity (for the theoretical basis of that approach see [10], Ch. 3.4). The log-likelihood function and its first-order derivatives associated with a sample (*X*_1_, …, *X*_*n*_) of i.i.d. observations from ${\left[F_{0}(\frac{\cdot \;-\;\mu }{\sigma })\right]}^{\theta }$ are readily obtained to be

$$\begin{array}{c} l\left(x_{1},\ldots ,x_{n};\theta ,\mu ,\sigma \right)=n\;\text{log}\;\theta +\left(\theta -1\right)\sum_{i=1}^{n}\text{log}\;F_{0}(\frac{x_{i}-\mu }{\sigma })+\sum_{i=1}^{n}\text{log}\;(\frac{1}{\sigma }f_{0}(\frac{x_{i}-\mu }{\sigma }))\;, \end{array}$$ (15)

$$\begin{array}{cc} \frac{\partial l}{\partial \theta } & =\frac{n}{\theta }+\sum_{i=1}^{n}\text{log}\;F_{0}(\frac{x_{i}-\mu }{\sigma }) \end{array}$$ (16.a)

$$\begin{array}{c} \frac{\partial l}{\partial \mu }=-\frac{\theta -1}{\sigma }\sum_{i=1}^{n}(\frac{f_{0}}{F_{0}})(\frac{x_{i}-\mu }{\sigma })-\frac{1}{\sigma }\sum_{i=1}^{n}(\frac{f_{0}^{\prime }}{f_{0}})(\frac{x_{i}-\mu }{\sigma }) \end{array}$$ (16.b)

$$\begin{array}{c} \frac{\partial l}{\partial \sigma }=-\frac{\theta -1}{\sigma }\sum_{i=1}^{n}(\frac{f_{0}}{F_{0}})(\frac{x_{i}-\mu }{\sigma })\cdot (\frac{x_{i}-\mu }{\sigma })-\frac{n}{\sigma }-\frac{1}{\sigma }\sum_{i=1}^{n}(\frac{f_{0}^{\prime }}{f_{0}})(\frac{x_{i}-\mu }{\sigma })\cdot (\frac{x_{i}-\mu }{\sigma }). \end{array}$$ (16.c)

Solving the corresponding system of score equations by means of the Newton-Raphson algorithm or an alternative numerical technique is an easy exercise, and the roots almost surely exist. The same cannot be said of the maximum likelihood estimator: examining the function (*θ*, *μ*, *σ*) ↦ *l*(*x*_1_, …, *x*_*n*_; *θ*, *μ*, *σ*) for various datasets revealed that it fails to attain almost surely a global maximum in the interior of the parameter space. Hence, carrying out a construction requiring that the MLE of *θ* exists and is asymptotically normal, is not practicable here.

In contrast to the MLE, the score statistic $U\left(\theta \right)\equiv l(x_{1},\ldots ,x_{n};\theta ,\tilde{\mu }\left(\theta \right),\tilde{\sigma }\left(\theta \right))$ with $\left(\tilde{\mu }\left(\theta \right),\tilde{\sigma }\left(\theta \right)\right)$ as the solution to $\frac{\partial l}{\partial \mu }=0$, $\frac{\partial l}{\partial \sigma }=0$ for fixed *θ*, is almost surely well defined. However, no approach to basing tests for interval hypotheses on a statistic of this form is at hand. An option for making use of *U*(*θ*) anyway for the purpose in mind, is to split up the equivalence testing problem (14) into the two one-sided testing problems

$$\begin{array}{c} H_{1l}:\left(\theta ,\mu ,\sigma \right)\in (-\infty ,\frac{1}{1+\varepsilon }]\times R\times R_{+}\;\text{vs.}\;K_{1l}:\left(\theta ,\mu ,\sigma \right)\in (\frac{1}{1+\varepsilon },\infty )\times R\times R_{+}\;, \end{array}$$ (17.1)

and

$$\begin{array}{c} H_{1r}:\left(\theta ,\mu ,\sigma \right)\in \left[1+\varepsilon ,\infty \right)\times R\times R_{+}\;\text{vs.}\;K_{1r}:\left(\theta ,\mu ,\sigma \right)\in \left(-\infty ,1+\varepsilon \right)\times R\times R_{+}\;. \end{array}$$ (17.2)

Rejecting *H*_1*l*_ and *H*_1*r*_ when it turns out that there holds $U\left(1/\left(1+\varepsilon \right)\right)/{\tilde{v}}_{l}>z_{1-\alpha }$ and $U\left(1+\varepsilon \right)/{\tilde{v}}_{r}<z_{\alpha }$, respectively, yields asymptotically valid tests for (17.1) and (17.2), provided ${\tilde{v}}_{l}^{2}$ consistently estimates the asymptotic variance of *U*(1/(1 + *ε*)) under *θ* = 1/(1 + *ε*) and ${\tilde{v}}_{r}^{2}$ that of *U*(1 + *ε*) under *θ* = 1 + *ε*. As usual, *z*_*γ*_ stands for the *γ*-quantile of N(0,1) for arbitrary *γ* ∈ (0, 1).

In order to avoid the possible inconsistency of the score test pointed out by D.A. Freedman [15], the variance estimators ${\tilde{v}}_{l}^{2}$ and ${\tilde{v}}_{r}^{2}$ should be based on the expected covariance matrix ***V***(*θ*, *μ*, *σ*), say, of the score statistics ${\left(\frac{\partial l}{\partial \theta },\frac{\partial l}{\partial \mu },\frac{\partial l}{\partial \sigma }\right)}^{\prime }$ evaluated at $\left(\theta ,\mu ,\sigma \right)=(1/\left(1+\varepsilon \right),\tilde{\mu }\left(1/\left(1+\varepsilon \right)\right),\tilde{\sigma }\left(1/\left(1+\varepsilon \right)\right))$ and $\left(\theta ,\mu ,\sigma \right)=(1+\varepsilon ,\tilde{\mu }\left(1+\varepsilon \right),\tilde{\sigma }\left(1+\varepsilon \right))$, respectively. No explicit expressions are available for the elements of ***V***(*θ*, *μ*, *σ*). However, the expected value of the random variables corresponding to the expressions appearing on the right-hand side of Eq (16.a-c) can be computed sufficiently fast and with high degree of accuracy by means of standard numerical integration techniques (like Gauss-Legendre quadrature), and the same holds true for the squares of these variables and thus also for the entries in ***V***(*θ*, *μ*, *σ*). Afterwards, ${\tilde{v}}_{l}^{2}$ and ${\tilde{v}}_{r}^{2}$ is computed as the reciprocal (1,1)-element of $V{\left(1/\left(1+\varepsilon \right),\tilde{\mu }\left(1/\left(1+\varepsilon \right)\right),\tilde{\sigma }\left(1/\left(1+\varepsilon \right)\right)\right)}^{-1}$ and $V{\left(1+\varepsilon ,\tilde{\mu }\left(1+\varepsilon \right),\tilde{\sigma }\left(1+\varepsilon \right)\right)}^{-1}$, respectively.

The last step of the construction consists in combining the two score tests for the one-sided problems (17.1) and (17.2) into a test for the two-sided equivalence problem (14) to be solved when interest is in establishing goodness-of-fit. The combined test rejects the null hypothesis *H* of (14) if and only if both of the critical inequalities $U\left(1/\left(1+\varepsilon \right)\right)/{\tilde{v}}_{l}>z_{1-\alpha }$ and $U\left(1+\varepsilon \right)/{\tilde{v}}_{r}<z_{\alpha }$ are found to hold true. According to a well-known and frequently exploited principle from the theory of equivalence testing (cf. [10, 16]; Ch. 7.1), the asymptotic validity of the one-sided tests with rejection regions $\left\{U\left(1/\left(1+\varepsilon \right)\right)/{\tilde{v}}_{l}>z_{1-\alpha }\right\}$ and $\left\{U\left(1+\varepsilon \right)/{\tilde{v}}_{r}<z_{\alpha }\right\}$ implies that the test for goodness-of-fit of a distribution from the family ${\left(F_{0}\left(\frac{\cdot \;-\;\mu }{\sigma }\right)\right)}_{\left(\mu ,\sigma \right)\in R\times R_{+}}$ carried-out in this way, is likewise asymptotically valid in terms of the significance level.

The facts stated so far admit the conclusion that in theory, testing for goodness-of-fit of distributional models involving unknown parameters is not an insurmountable challenge for statistical inference. However, upon studying the power of such a test, this judgement can hardly be maintained: Determining the rejection probability of the double one-sided score-test procedure described above by means of Monte Carlo simulation of normally distributed data reveals that even against the null alternative of perfect fit of the model [⇔ *θ* = 1] and for a choice of the equivalence margin of moderate strictness [*ε* = 0.5077, corresponding to a maximum acceptable vertical distance of the cdf’s of *δ* = 0.15—recall Table 1], several thousands (!) of observations are required in order to attain a power of 80%. Thus, establishing goodness-of-fit of some distributional shape rather than a specific element of the corresponding family of distributions (like $N(\mu ,\sigma^{2}))$ by means of a testing procedure providing satisfactory control over both kinds of error-risk, is hardly an option for practice.

## 6 Illustrating example

**Fig 2:**
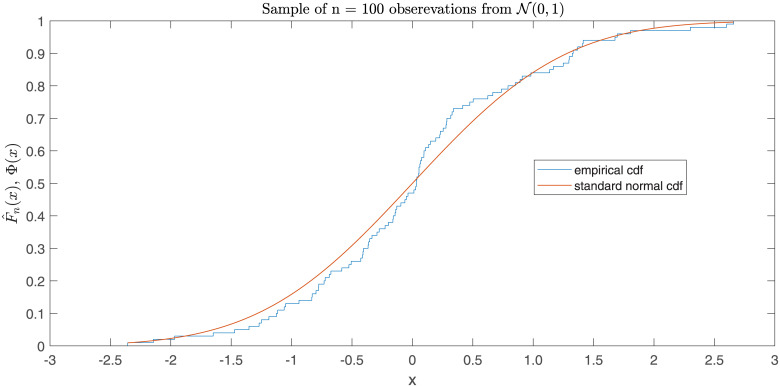
Plot of the empirical cdf computed from the dataset S1 Dataset appended as supporting information, and the standard normal cdf Φ as the model to be established.

Fig 2 shows the plot of the empirical cdf of a simulated random sample of size *n* = 100 from N(0,1), together with the theoretical cdf Φ to be assessed for goodness-of-fit to these data.

In order to apply the UMP test of Section 3 with *F*_0_ = Φ, the maximum likelihood estimate of the Lehmann parameter *θ* has to be determined. With the data behind the empirical cdf plotted above and *F*_0_ = Φ, evaluating Eq (6) yields ${\hat{\theta }}_{n}\left(F_{0}\right)$ = 1.0793. If the equivalence margin for *θ* is chosen to be *ε* = 0.5077, it is seen from Table 2 that the observed value of ${\hat{\theta }}_{n}\left(F_{0}\right)$ has to be checked for inclusion in the interval (0.7883, 1.2887). Since 1.0793 is an inner point of this interval, the conclusion is that the goodness-of-fit test for standard normality at level *α* = 0.05 leads under the chosen specifications to rejecting the null hypothesis of lack-of-fit.

Assuming next that the distribution one wants to fit is $N(\mu ,\sigma^{2})$ with both parameters being unknown and keeping the equivalence limits for *θ* set at 1/(1 + *ε*) and 1 + *ε* with *ε* = 0.5077, carrying out the double one-sided score test described in Section 5 has to start with computing the restricted MLE’s of (*μ*, *σ*) given *θ* = 1/(1 + *ε*) and *θ* = 1 + *ε*. From the dataset under analysis, these estimates are obtained to be $\left(\tilde{\mu },\tilde{\sigma }\right)\left(1/\left(1+\varepsilon \right)\right)$ = (0.3760, 0.8434) and $\left(\tilde{\mu },\tilde{\sigma }\right)\left(1+\varepsilon \right)$ = (−0.3308, 1.0636). Calculating the corresponding efficient scores of *θ* gives *U*(1/(1 + *ε*)) = 0.4444 and *U*(1 + *ε*) = 2.2406, with estimated asymptotic variances ${\tilde{v}}_{l}^{2}$ = 1.636889 and ${\tilde{v}}_{r}^{2}$ = 0.276968. Finally, standardizing the two score statistics yields $U\left(1/\left(1+\varepsilon \right)\right)/{\tilde{v}}_{l}$ = 1.751258 and $U\left(1+\varepsilon \right)/{\tilde{v}}_{r}$ = 0.844329 of which only the first one leads to rejecting the corresponding one-sided null hypothesis whereas $U\left(1+\varepsilon \right)/{\tilde{v}}_{r}$ is much larger than *z*_*α*_ = −1.64485. Hence, testing for normal shape of the distribution underlying the dataset fails to lead to a decision in favor of the alternative hypothesis of the corresponding equivalence problem (14).

## 7 Discussion

As major strengths of the procedure obtained in the core of this paper for testing for goodness-of-fit of a fully specified distribution of the continuous type to the distribution underlying a given random sample, the following facts can be adduced:

(i) The alternative hypothesis which can be declared established upon a positive result, states that the model fits the data sufficiently well rather than meriting rejection because of marked discrepancies from the true distribution.
(ii) The method is fully exact and satisfies the strongest of the optimality criteria having been in use for hypothesis tests since the beginnings of classical frequentist inference.
(iii) Due to the availability of a fairly compact source code both in SAS/IML and R, the practical implementation of the test, as well as the algorithm for exact power and sample size computation, is fast and easy.
(iv) The primary metric, in terms of which the region of distribution functions defined equivalent to the distribution function specified by the model, is fully intuitive also for applied research workers without advanced statistical training. It is the same in terms of which alternatives to the null hypothesis of perfect fit of the model are expressed in the Kolmogorov test.

Admittedly, the last of these advantages comes into play only as long as one is willing to rely on the semiparametric model which assumes that the true distribution function underlying the data differs from that specified by the model through taking all values of the latter to the *θ*-th power. Except for ignoring right-censoring and applying the transformation *u* ↦ *u*^*θ*^ to cumulative distribution rather than survivor functions, this model is the same as Cox’s [13] well-known proportional hazards model. If the proposed test does not lead to a decision in favor of equivalence between actual and hypothesized distribution, one cannot rule out the possibility that the true distribution differs from *F*_0_ nowhere by more than a given margin *δ* without satisfying the modified Cox model.

## Supporting information

S1 File (R)

S2 File (SAS)

S1 Dataset Sample used for illustration. (XLSX)
